# Comprehensive analysis reveals signal and molecular mechanism of mitochondrial energy metabolism pathway in pancreatic cancer

**DOI:** 10.3389/fgene.2023.1117145

**Published:** 2023-02-06

**Authors:** Hong Yang, Ye Cui, YuMing Zhu

**Affiliations:** ^1^ Department of Hepatobiliary and Pancreatic Surgery, The Third Affiliated Hospital of Chongqing Medical University (Gener Hospital), Chongqing, China; ^2^ Beijing GAP BioTechnology, Beijing, China

**Keywords:** mitochondria, pancreatic cancer, gene set enrichment analysis, prognosis model, immunotherapy

## Abstract

Pancreatic cancer (PAAD) is one of the most malignant tumors with the worst prognosis. The abnormalities in the mitochondrial energy metabolism pathway are intimately correlated with the occurrence and progression of cancer. For the diagnosis and treatment of pancreatic cancer, abnormal genes in the mitochondrial energy metabolism system may offer new targets and biomarkers. In this study, we compared the dysregulated mitochondrial energy metabolism-associated pathways in PAAD based on pancreatic cancer samples in the Cancer Genome Atlas (TCGA) database and normal pancreas samples from the Genotype Tissue Expression project (GTEx) database. Then identified 32 core genes of mitochondrial energy metabolism pathway-related genes (MMRG) were based on the gene set enrichment analysis (GSEA). We found most of these genes were altered among different clinical characteristic groups, and showed significant prognostic value and association with immune infiltration, suggesting critical roles of MMRG involve tumor genesis of PAAD. Therefore, we constructed a four-gene (LDHA, ALDH3B1, ALDH3A1, and ADH6) prognostic biomarker after eliminating redundant factors, and confirming its efficiency and independence. Further analysis indicated the potential therapeutic compounds based on the mitochondrial energy metabolism-associated prognostic biomarker. All of the above analyses dissected the critical role of mitochondrial energy metabolism signaling in pancreatic cancer and gave a better understanding of the clinical intervention of PAAD.

## Introduction

Pancreatic cancer has an extremely high mortality rate and is one of the most frequent causes of death from cancer (Raimondi et al., 2009; Wang-Gillam et al., 2022). Only 4% of people with pancreatic cancer survive 5 years following diagnosis, despite improvements in its detection and treatment (Petersen et al., 2006; Vincent et al., 2011; Erkan et al., 2012). 80%–85% of patients cannot remove cancer variants through surgery in an advanced stage (Klein, 2021). Additionally, the majority of chemotherapeutic drugs do not effectively treat pancreatic cancer. Polymorphic genes may interact with other genes or environmental variables to cause some spread of pancreatic tumors (Raimondi et al., 2009; Yang et al., 2021). Mitochondria, known as the energy source of cells, represents the key intracellular signal transduction center and is an important determinant in the genesis and progression of cancer, affecting metabolic reprogramming, the development of metastatic capacity, and response to chemotherapy drugs (Guerra et al., 2017; Kalyanaraman et al., 2018; Ji et al., 2022). Abnormal mitochondrial pathways and metabolic disorders can lead to changes in gene expression, thereby promoting the development and progression of cancer and immune system escape (Egan et al., 2021; Ye et al., 2021). Therefore, we need to understand the impact and potential role of the mitochondrial energy metabolism pathway in the occurrence and development of pancreatic cancer.

Most of the energy required by aerobic cells to maintain their physiological functions is produced by mitochondria, which are the primary sites of oxidative phosphorylation and ATP production. Additionally, they take involved in apoptosis, information transfer, differentiation, and cell proliferation. (Carew and Huang, 2002; Boese and Kang, 2021). Mitochondrial energy metabolism can be brought on by a wide range of conditions affecting the mitochondria, including oxidative stress, abrupt changes in ion concentration, abnormal oxidative phosphorylation, changes in the electron transfer chain complex enzymes, and mutations in the mitochondrial DNA. (Ji et al., 2022; Shen et al., 2022). In many cancer cells, bioenergy reprogramming involves the transformation of maximum ATP generated by oxidative phosphorylation (OXPHOS) from static and differentiated cells to the requirement of balancing energy demand and substrate generation in fast-growing cells to achieve cell biogenesis and reproduction (Wallace, 2012; Guerra et al., 2017; Egan et al., 2021; Shen et al., 2022). Cancer and mitochondrial energy metabolism are intimately connected. For example, the resistance of breast cancer cells to PI3K inhibition is associated with the transformation from glucose to lactic acid (Park et al., 2016). The transition of ovarian cancer cells from OXPHOS to glycolysis is accompanied by an increase in antioxidant defense that seems to be dependent on the pentose phosphate pathway (PPP). (Catanzaro et al., 2015), and glycolysis are highly differentially expressed in melanoma (Falkenius et al., 2013). Additionally, the expression of the β-F1-ATP enzyme is specifically suppressed in renal and colon cancer, whereas glycolytic glyceraldehyde-3-phosphate dehydrogenase expression was elevated (Cuezva et al., 2002). The progression of a tumor is indicated by mitochondria based on the bioenergy features of cancer. Targeting mitochondria in immune cells and glycolytic bioenergy and metabolism may currently be an effective therapeutic approach for the management of cancer. The significance of investigating novel therapeutic approaches is underscored by the distinct mitochondrial and metabolic biology of these cancer cells.

In this study, we systematically analyzed the mitochondrial energy metabolism pathway significantly dysregulated in pancreatic cancer in the TCGA database and excavated the core genes related to the mitochondrial energy metabolism pathway. To comprehensively describe the impact of core genes related to the mitochondrial energy metabolism pathway on the occurrence and development of pancreatic cancer, we analyzed mutation, expression level, survival analysis, protein interaction, and the correlation of biological tumor immune microenvironment. In addition, a prognostic risk-scoring model was constructed and validated in the validation set. Based on the mitochondrial energy metabolism pathway signature, it can effectively predict the prognosis and therapeutic effects of patients, and screen potential therapeutic compounds for pancreatic cancer.

## Materials and methods

### Data collection and processing

Expression profiles of pancreatic cancer patients and normal control samples from TCGA and GTEx database respectively, were obtained from the UCSC Xena resource (https://xenabrowser.net/datapages/). These data contained 181 samples from TCGA (177 cancer samples and 4 normal samples) and 167 normal samples from the GTEx database. The publicly available copy number variation (CNV) and single-nucleotide variation (SNV) data of TCGA-PAAD cohorts were downloaded from the TCGA database based on the R package *TCGAbiolinks*. Clinical characteristics (including age, gender, cancer grade, chemotherapy response, history of chronic pancreatitis, smoking status, diabetes history and alcohol consumption status, overall survival (OS), etc.) of TCGA-PAAD samples were also obtained and shown in Table 1 (*N* = 176, 1 case with no complete survival information was removed). The GEO database (https://www.ncbi.nlm.nih.gov/geo) was used to gather the expression profile and clinical information of the independent validation cohort (GSE79668, *N* = 51). For immunotherapy response analysis, we obtained the expression data and detailed clinical information of 298 metastatic urothelial cancer who were treated with anti-PD-L1 agent (IMvigor210 cohort) based on R package IMvigor210CoreBiologies (Mariathasan et al., 2018). Also, we collected the expression data and detailed clinical information of 97 melanoma patients treated with immune checkpoint blockade (GSE91061) for further evaluation. Besides, global protein-protein interaction (PPI) network from STRING database (https://www.string-db.org) was obtained to analysis the potential gene interactions. STRING database provided both validated and predicted interactions for MMRG core genes, including direct (physical) and indirect (functional) associations.

**TABLE 1:** Clinical characteristics of pancreatic cancer patients.

Characteristic	Patients (176)
Radiation therapy	
F	101 (76%)
T	32 (24%)
Unknown	43
History of chronic pancreatitis	
F	13 (9.3%)
T	127 (91%)
Unknown	36
History of diabetes	
F	107 (74%)
T	38 (26%)
Unknown	31
Alcohol history documented	
F	64 (39%)
T	100 (61%)
Unknown	12
Tobacco smoking history	
1	65 (45%)
2	20 (14%)
3	28 (20%)
4	23 (16%)
5	7 (4.9%)
Unknown	33
Vital status	
Alive	118 (67%)
Dead	58 (33%)
Grade	
Grade1/2	124 (71%)
Grade3/4	50 (29%)
Unknown	2
Age	
<60	54 (31%)
≥ 60	122 (69%)
Gender	
FEMALE	80 (45%)
MALE	96 (55%)
Risk score	
Low	88 (50%)
High	88 (50%)

### Acquisition of MMRG core genes, differential expression analysis and survival analysis

Mitochondrial energy metabolism-associated pathways were collected according to a previous study by (Ye et al., 2021). The corresponding genes involved in these pathways (termed mitochondrial energy metabolism pathway-related gene (MMRG)) were retrieved from the KEGG database of MSigDB (https://www.gsea-msigdb.org/gsea/msigdb). Data from the TCGA-PAAD and GTEx project was batch-corrected and normalized according to the previous process (https://github.com/mskcc/RNAseqDB) (Wang et al., 2017). GSEA analysis was performed to identify the dysregulated mitochondrial energy metabolism-associated pathways between PAAD and normal tissues. The differential expression analysis between normal and malignant tissues was investigated using the R package limma. And the core enrichment genes identified by the GSEA analysis based on R package *clusterProfiler* were defined as MMRG core genes. The differential expression of these genes between different clinical characteristics groups from the TCGA-PAAD cohort (including grade, stage, drinking, smoking, history of chronic pancreatitis, history of diabetes, cancer location, gender, age, radiotherapy or not, chemotherapy response) were compared by Wilcoxon rank-sum test. To investigate the prognostic value of MMRG core genes, samples were split by median expression of each gene, and OS was compared by log-rank test.

### Construction of a prognostic risk model for pancreatic cancer

The hazard ratio (HR) and prognostic significance of genes were assessed using univariable cox regression analysis, and genes with *p* < 0.05 were screened for prognostic relevance. LASSO regression analysis further screened prognostic factors (R package glmnet), and the expression from each prognostic factor was weighted with the LASSO regression coefficient to create a risk score model for predicting sample survival:

$$Score=\overset{n}{\underset{i=0}{\sum }}\beta i*\chi i$$

Where βi represents the corresponding weight coefficient of each gene, χi represents the expression of each gene, and n represents the number of signature genes. Based on the median score, the sample was split into high- and low-risk groups. Subsequently, Kaplan-Meier survival curves were created for prognostic analysis, and any differences were then analyzed using log-rank tests to determine significance. The receiver operating characteristic (ROC) curve was employed to assess the prediction of scoring by the risk score model, and the R package survivalROC was used to display the area under the curve (AUC). To further verify the prediction model, the GSE79668 dataset was employed.

### Analysis of immune cell infiltration levels

Immune and stromal scores of TCGA-PAAD samples were computed using the ESTIMATE method based on particular gene expression profiles of immune and stroma cells. Additionally, we also estimated the immune cell infiltration based on three different methods described below: 1) CIBERSORT, an analytical tool to characterize the abundance of cell types. 22 immune cell types, containing natural killer (NK) cells, naive and memory B Cells, myeloid subpopulations, plasma cells, and 7 different types of T Cells, were identified using the leukocyte signature gene matrix LM22, which consists of 547 genes. The proportions of the 22 cell phenotypes in the samples were calculated by CIBERSORT in conjunction with the LM22 feature matrix, and for each sample, the proportions of all estimated immune cell types added up to 1. 2) Single sample gene set enrichment analysis (ssGSEA) was implemented to determine the proportion of 28 immune cell infiltrates by R package GSVA. 3) The 64 immune cell infiltration proportions were calculated by the R package xCell.

### Drug resistance analysis and screening of potential therapeutic compounds

We first predicted the sensitivity of TCGA-PAAD patients to drugs in the GDSC v. 2 database (https://www.cancerrxgene.org/) using the R package *oncoPredict*. Then the spearman correlation between log-transformed half maximal inhibitory concentration (IC50) for each drug and risk score was calculated. The PDB database (https://www.rcsb.org/) was used to download information on protein structure (PDB ID: 5ZJF), while the ZINC small molecule database (https://zinc.docking.org/) was employed to access data of cisplatin compound (Cisplatin, ZINC6507066). Water molecules were removed by AutoDockTools-1.5.6 software, and then exported to the pdbqt format for docking after hydrogenation. The mol2 format of Cisplatin structure was converted to pdbqt format by anaconda, OpenBabel-3.1.1 and MGLTools-1.5.6. Afterwards, autodock-vina was used for molecular docking, and the software PyMOL was used for protein-compound binding maps.

### Statistical analysis

The Wilcoxon rank-sum test was used to compare significant differences between two groups of samples (including gene expression, immune cell infiltration), while Kruskal–Wallis test was used to compare significant differences among over than two groups of samples. The threshold for statistical significance was *p* < 0.05. In the study, ns stands for *p* > 0.05, * for *p* < 0.05, ** for *p* < 0.01, *** for *p* < 0.001, and **** for *p* < 0.0001.

## Results

### MMRG core gene and mutation status in pancreatic cancer

**FIGURE 1:**
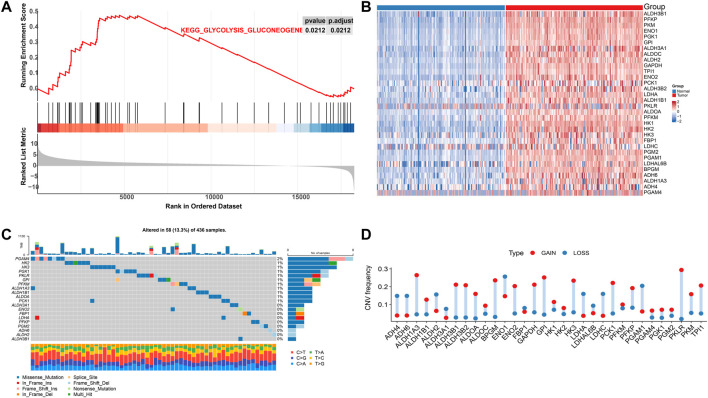
Analysis of MMRG gene function and core gene mutation status in pancreatic cancer. **(A)** Disordered mitochondrial energy metabolism-related pathways in pancreatic cancer; **(B)** Heat map of core genes in significant gene concentration; **(C)** Mutation waterfall diagram of MMRG gene set; **(D)** CNV mutation frequency distribution map.

Five pathways of the mitochondrial energy metabolism (KETONE_BODY_METABOLIC, OXIDATIVE_PHOSPHORYLATION, FATTY_ACID_METABOLISM, GLYCOLYSIS_GLUCONEOGENESIS, and CITRATE_CYCLE) were analyzed by GSEA enrichment. The findings revealed that only the GLYCOLYSIS_GLUCONEOGENESIS pathway was significantly enriched in the pancreatic cancer data (Figure 1A). These results suggested changes in mitochondrial energy metabolism in PAAD. Extraction of its core genes yielded 32 genes as mitochondrial energy metabolism pathway-related genes MMRG, which were significantly differentially expressed in normal and tumor samples (Figure 1B). It indicated that the MMRG core gene played an important role in PAAD. We further investigated the genomics alteration of MMRG core genes. For somatic mutation, we observed that most of the MMRG core genes had few mutations. The most mutated gene was PGAM4 (2%) and it had mainly missense mutations (Figure 1C). For copy number variation of the MMRG core genes, the amplified events were predominant (Figure 1D).

### Differential expression of MMRG core genes in clinical features

**FIGURE 2:**
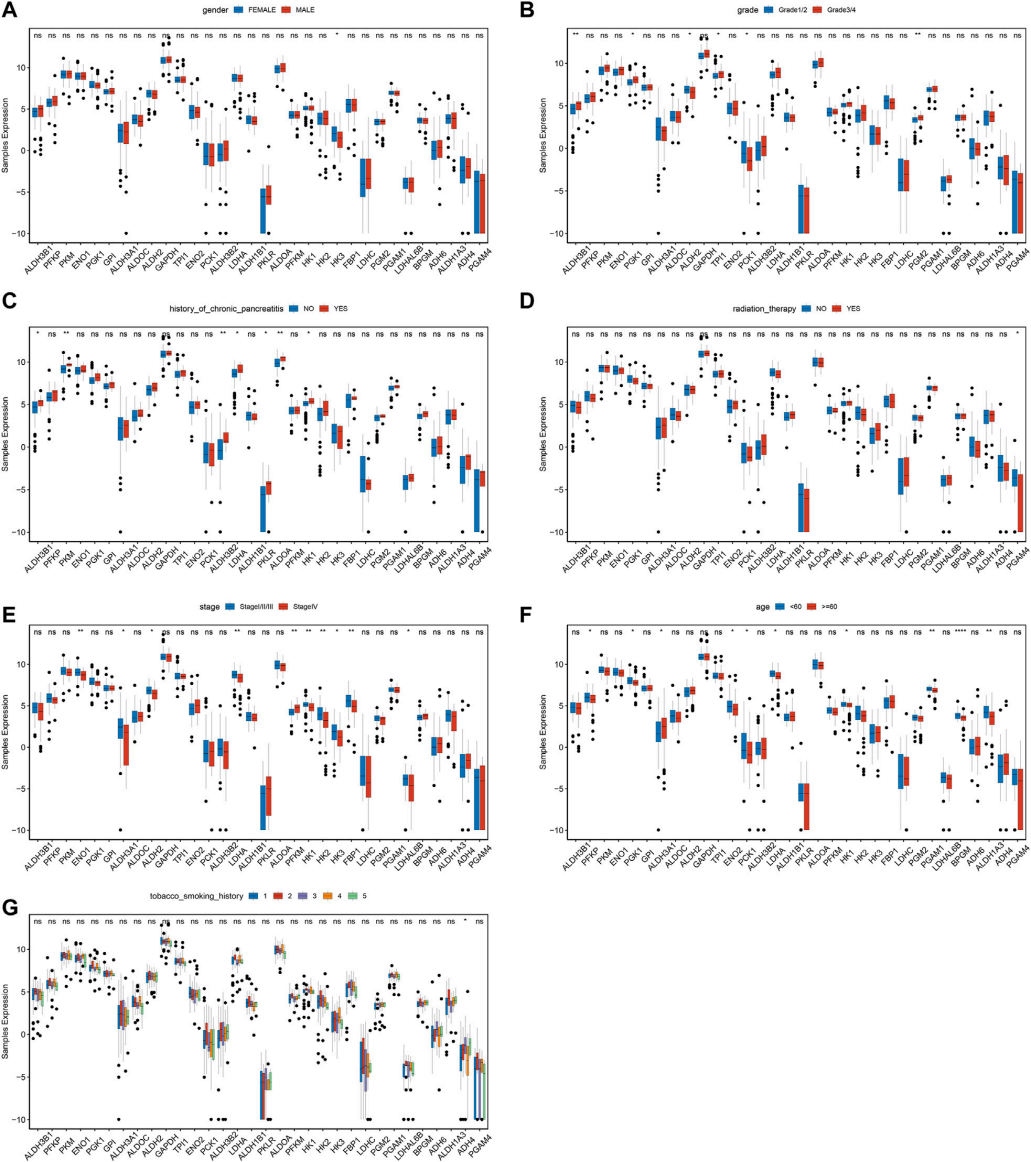
Expression difference of MMRG core gene of clinical characteristics in tumor and normal samples. **(A–G)** The expression difference of MMRG core gene set of gender, grade, history of chronic pancreatitis, radiotherapy, stage, age, and smoking history, respectively.

To further investigate the differences of MMRG core gene in pancreatic cancer among different clinical characteristics groups, the expression status of gender, grade, history of chronic pancreatitis, radiation therapy, stage, age, and history of tobacco smoking were analyzed. We found significant differences in HK3 between genders (Figure 2A, *p* < 0.05). ALDH3B1, ENO1, ALDH2, GAPDH, and LDHC had significant differences among grades (Figure 2B), while ALDH3B1, PKM, and ALDH3B2 differed among chronic pancreatitis histories (Figure 2C). Meanwhile, PGAM4 showed significant differences among different reflex treatments (Figure 2D). For ENO1, ALDH3A1, and ALDH2, significant changes exist between stages (Figure 2E). Other genes, including PFKP, PGK1, and ALDH3A1 fluctuated significantly with age (Figure 2F), and ADH4 differed significantly with smoking history (Figure 2G). The findings demonstrated that various clinical characteristics of PAAD were influenced by the MMRG core gene.

### Survival analysis of MMRG core gene in pancreatic cancer

**FIGURE 3:**
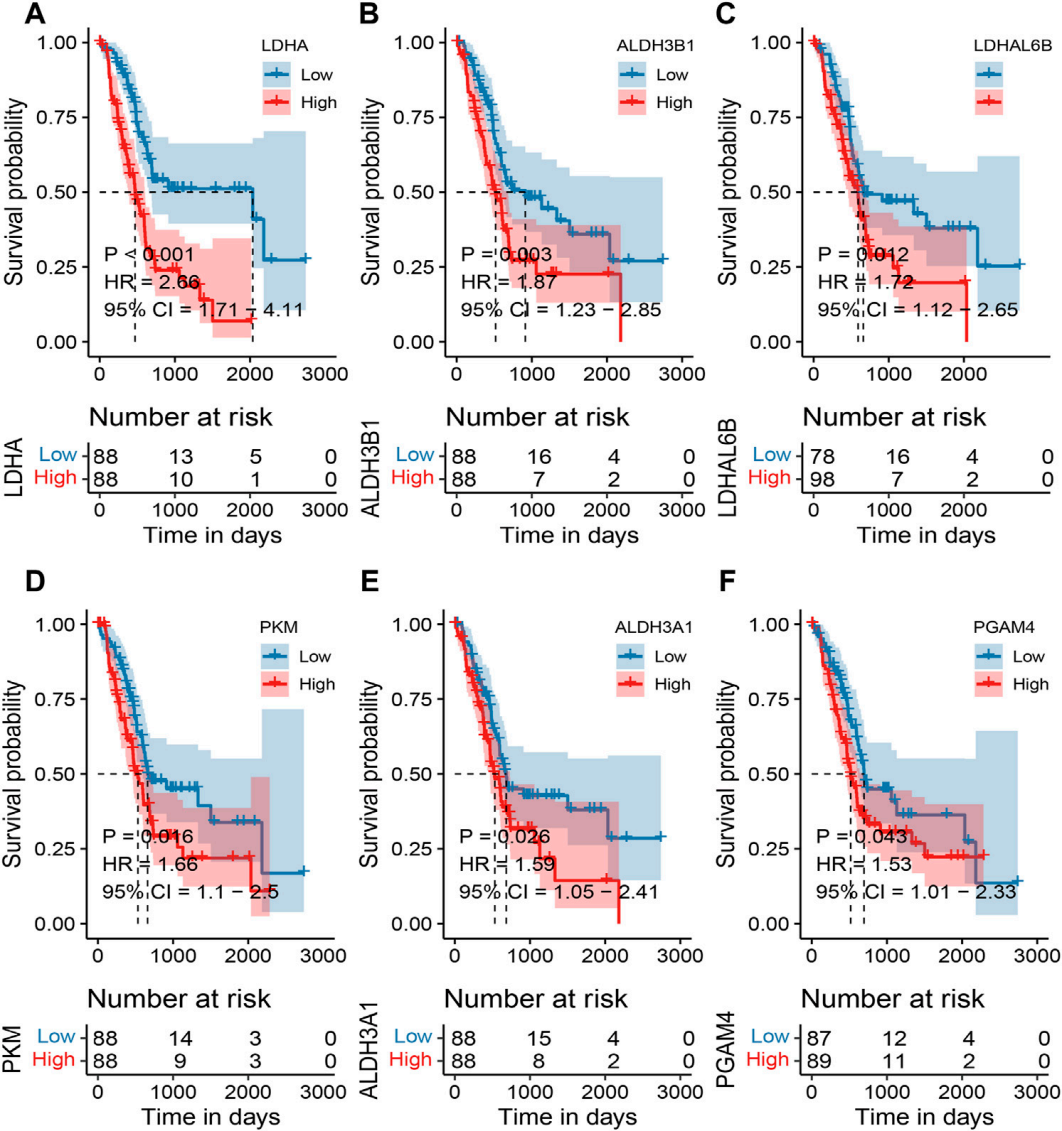
Survival analysis of MMRG core gene in pancreatic cancer patients. **(A–F)** Survival analysis of LDHA, ALDH3B1, LDHAL6B, PKM, ALDH3A1, and PGAM4, respectively (*p* < 0.05).

Based on the TCGA expression and survival data, the OS differences between the high and low expression of MMRG core genes were analyzed, and high and low expression groups were split according to the median expression value. 6 differentially expressed MMRGs (LDHA, ALDH3B1, LDHAL6B, PKM, ALDH3A1, and PGAM4) exhibited survival significance, as demonstrated by the survival analysis of 32 MMRGs in pancreatic cancer (Figures 3A–F, *p* < 0.05). Patients with pancreatic cancer in the low-expression group had a much higher survival rate than those in the high-expression group, with the LDHA associated with glycolysis being the most significant (*p* < 0.001).

### The immune microenvironment of MMRG core genes

**FIGURE 4:**
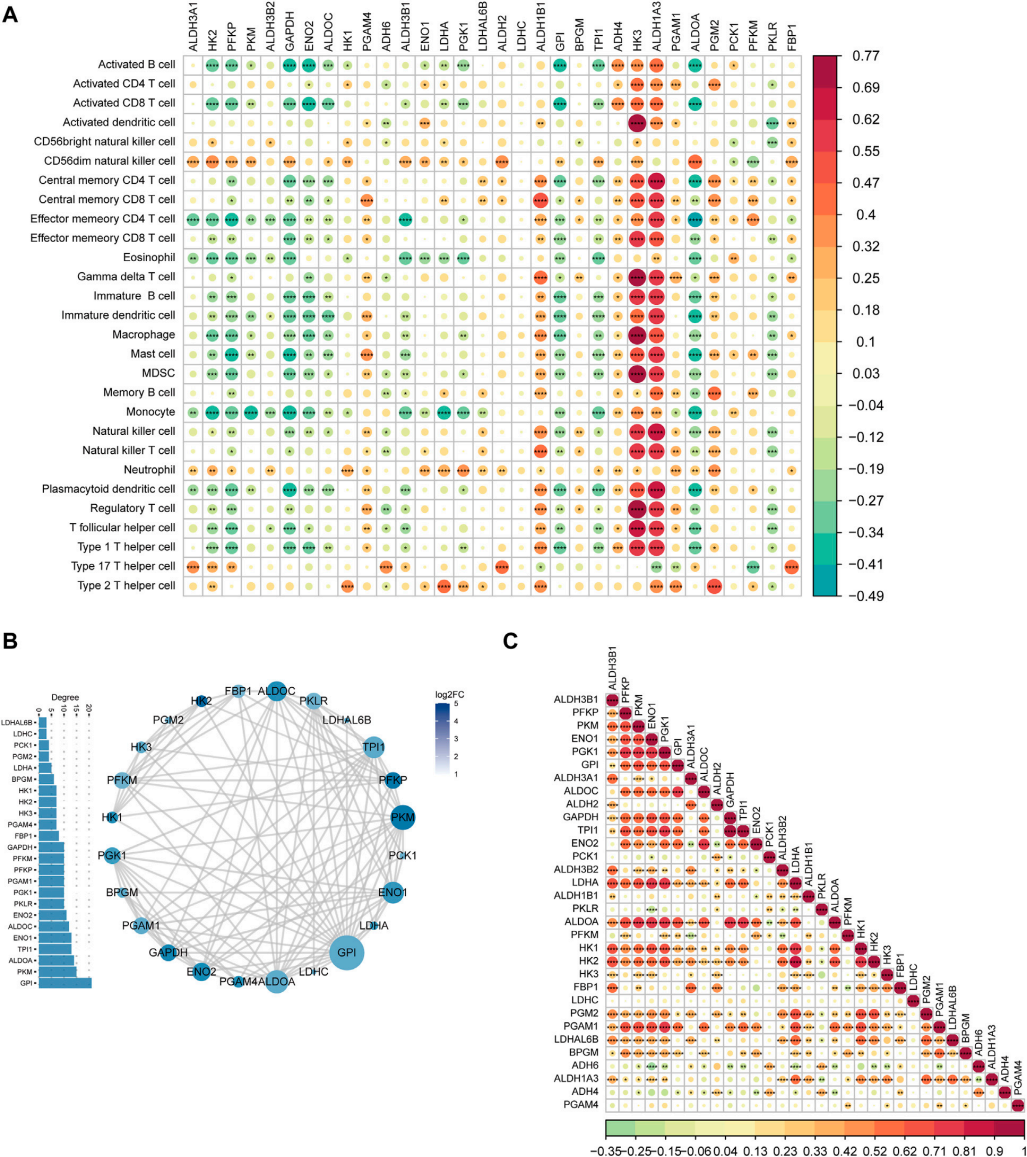
MMRG core gene immune microenvironment and protein-protein interaction (PPI) network. **(A)** The correlation between differential expression of MMRG and tumor immune cell infiltration (positive in red and negative in blue); **(B)** MMRG core gene protein-protein interaction (PPI) network. Color of the gene indicates the differential expression (log2FC) and the node size indicates the degree which also displayed by the barplot leftside; **(C)** Spearman correlation of MMRG core gene.

To understand the contribution of MMRG core genes on immune response, we first calculated the correlation between MMRG core genes expression and immune cell infiltration. Most of the expression levels of MMRG were associated with the infiltration of various immune cells in the tumor immune microenvironment. In particular, HK3, ALDH1A3, and ALDH1B1 showed significant positive association with the infiltration of most immune cell types (Figure 4A, *p* < 0.05). This is consistent with previous research that HK3 is correlated with immune infiltrates and predicts response to immunotherapy (Tuo et al., 2020; Xu et al., 2021). Also, highly expressed ALDH1A3 played an important role in immune response of tumor (Samson et al., 2019). On the contrary, HK2, GAPDH, PFKP, ENO2 showed predominant negative association with immune infiltration (Figure 4A, *p* < 0.05). HK2 contributes to immune responses and is upregulated during inflammation (Hinrichsen et al., 2021). While GAPDH modulates immunity through metabolism-associated pathways (Kornberg et al., 2018). These results suggested that MMRG core genes may participate in the process of tumor immune invasion.

### MMRG core genes interaction analysis

Based on the STRING database, we investigated the potential gene interactions of these MMRG core genes which played critical roles in glycolysis of mitochondrial energy metabolism. Within the PPI network, gene nodes such as GPI, PKM, ALDOA, and TPL1 were highly connected and might play a dominant role in the reciprocal relationship (Figure 4B). In addition, the spearman correlation analysis among the MMRG core genes (Figure 4C) showed that there was a significant positive correlation between TPL1 and GAPDH (*p* < 0.05, corr = 0.94), LDHA and HK2 (*p* < 0.05, corr = 0.83).

### MMRG core gene signature construction and prognostic analysis

The univariable cox risk regression model was constructed by the 32 obtained MMRG core genes as candidate genes. 14 potential prognostic factors were identified according to the threshold *p* < 0.05 (Supplementary Figure S1AB). Then, the genes used to develop the risk model were further investigated using LASSO Cox regression analysis. Four key prognostic genes were assessed after redundant factors and overfitting were eliminated: LDHA, ALDH3B1, ALDH3A1, and ADH6 (Supplementary Figures S1B, C). To create a survival risk score model for PAAD samples, the expression of these four genes was weighted along with the LASSO regression coefficients.

**FIGURE 5:**
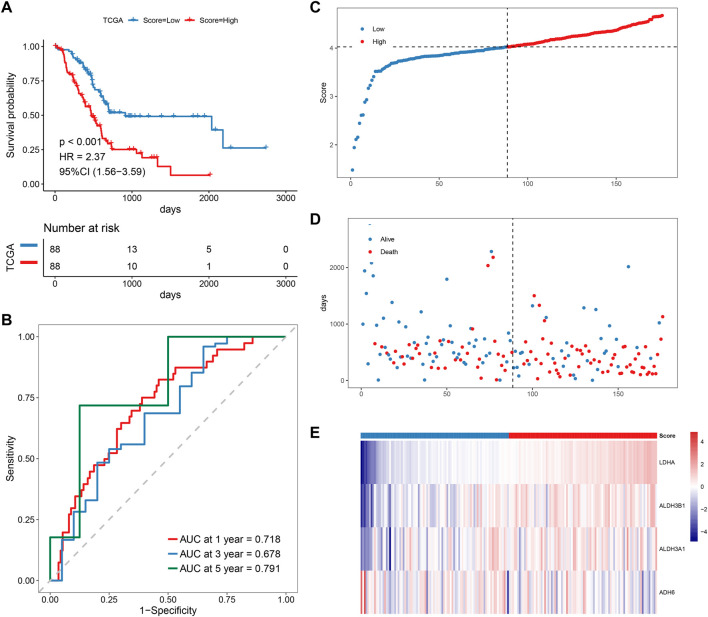
Prognostic analysis of MMRG core gene signature. **(A–E)** KM, ROC, risk score, survival time distribution, and expression of prognostic factors of MMRG core gene signature.

Patients with PAAD were divided into high-risk and low-risk groups based on the predicted risk scores and the median score. According to the survival analysis, both the training set and the validation set had a better prognosis for the low-risk score group (Figure 5A). The sensitivity and specificity of the signature were assessed by time-related ROC analysis. The AUC values were 0.718, 0.678, and 0.791 for one, three, and 5 years for the TCGA cohort (training set), while 0.633, 0.761, and 0.932 for the GSE79668 cohort (validation set), respectively (Figure 5B, Supplementary Figure S2B). Comparing the score distributions of the two groups, it can be found that the score distributions of both the training and validation sets were more continuous and devoid of outliers or extremes (Figure 5C, Supplementary Figure S2C). In both the training and validation sets, the survival pattern was consistent, and the low-risk group performed better (Figure 5D, Supplementary Figure S2D). Finally, the training and validation sets of the prognostic factor expression model demonstrated similar gene expression trends (Figure 5E, Supplementary Figure S2E).

### Correlation of risk score models and clinical characteristics

**FIGURE 6:**
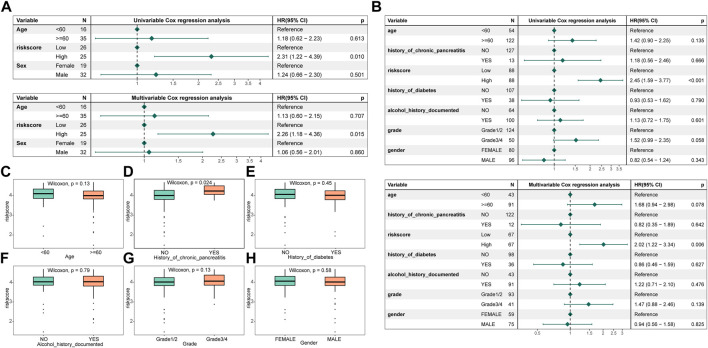
Risk assessment model construction and correlation of clinical characteristics with different groups. **(A)** Forest map of univariable and multivariate cox factors in the training set; **(B)** Forest map of univariable and multivariate cox factors in the validated set; **(C–H)** Risk scores in different clinical characteristics (age, history of chronic pancreatitis, history of diabetes, history of alcohol documented, grade, and gender, respectively).

To ascertain whether the signature created was an independent prognostic predictor, both univariable and multivariate cox analyses were used. by a threshold *p* < 0.05. The results exhibited that the signature functioned as an independent prognostic factor in the training and validation set data (Figures 6A, B). By comparing the distribution of risk score subgroups across clinical characteristics, we were able to further investigate the independence of the model. Subgroups with sample sizes of 2 and below were excluded from the analysis. The study revealed that there was a substantial difference in the clinical data on the history of chronic pancreatitis across the groups (Figures 6C–H).

### Correlation of immune infiltration in different risk subgroups

**FIGURE 7:**
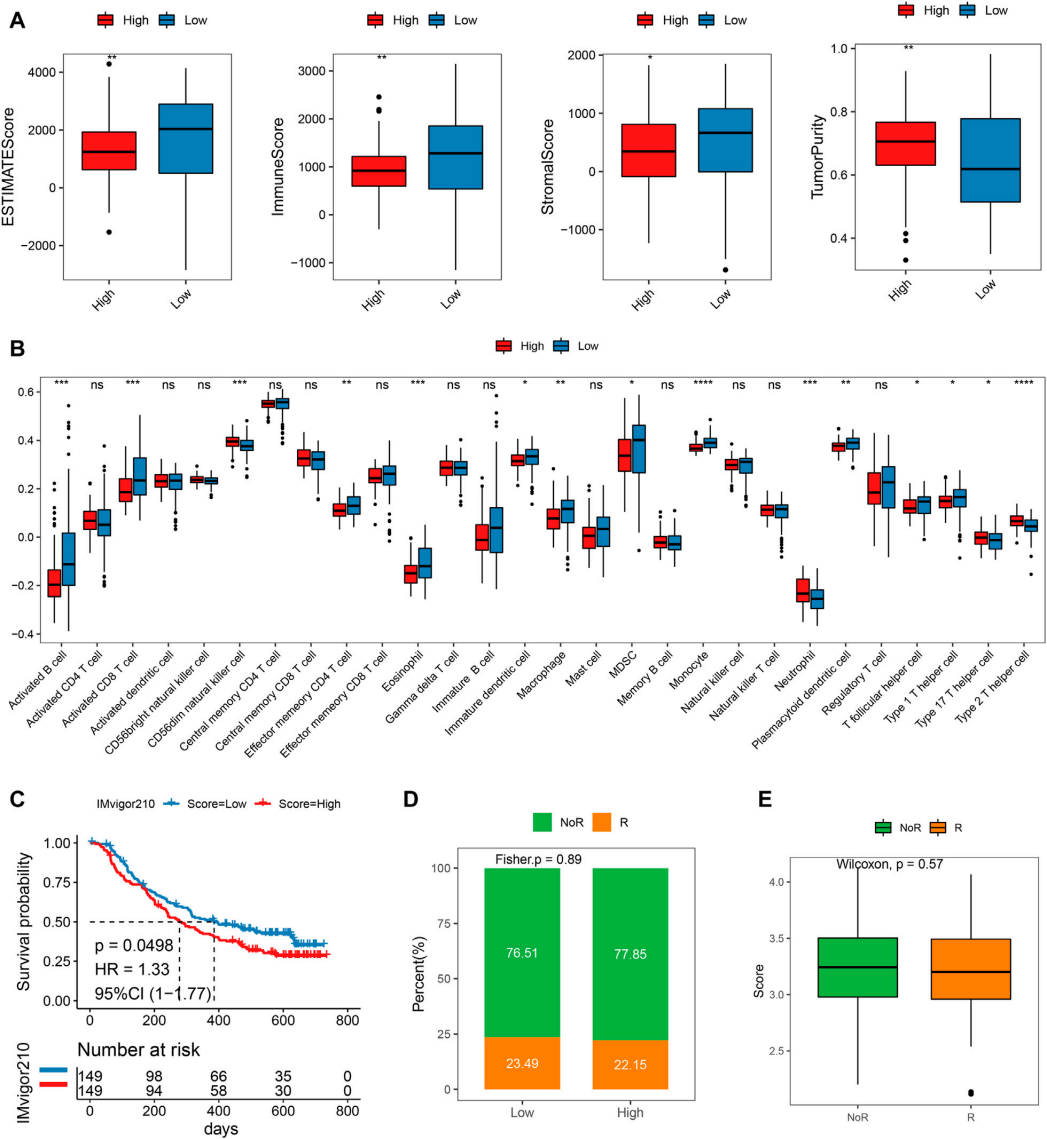
Difference of immune infiltration and prognosis of immunotherapy between high and low-risk scoring groups. **(A)** ESTIMATEScore, ImmuneScore, StromalScore, TumorPurity of high and low score groups; **(B)** Immune infiltration score of high-risk groups; **(C)** KM curve of the high or low-risk groups; **(D)** Objective response rate in high- and low-risk groups; **(E)** Risk score distribution in different clinical response groups.

The ESTIMATE score of the low-risk subgroup was much greater than that of the high-risk subgroup, as illustrated in Figure 7A. (*p* < 0.01). Additionally, the immunological and stromal scores of the low-risk subgroup were both considerably higher than those of the high-risk subgroup (*p* < 0.05). When compared to the low-risk grouping, the tumor purity of the high-risk subgroup was noticeably higher. The results illustrated that the prognosis was better in the low-risk subgroup, which was also in line with our above findings obtained. The considerable variations in immune cell infiltration scores between the high- and low-risk groups were evident from the disparities in immune infiltration scores. For example, the high-risk group had significantly higher immune infiltration ratings for CD56dim neutrophils, natural killer cells, and type 2T helper cells compared to the low-risk group (*p* < 0.05). However, the immune infiltration scores of low-risk molecules were significantly higher in 10 cell types such as activated B Cell, activated CD8 T Cell, and macrophage, etc. (Figure 7B, *p* < 0.05).

### Potential therapeutic strategies for MMRG

We then pretend to observe the predictive performance of our mitochondrial energy metabolism-associated prognostic biomarker in immunotherapy patients. Since publicly available PAAD immunotherapy response data are not available, we employed datasets from other cancer types instead, to investigate the generalizability of our mitochondrial energy metabolism-associated prognostic biomarker. Risk scores of metastatic urothelial cancer patients treated with the anti-PD-L1 agent (IMvigor210 cohort, *N* = 298) were predicted according to the original formula, and patients were grouped by median risk score. The comparison showed the low-risk group had a better prognosis (log-rank test *p* = 0.0498, Figure 7C). But the objective response rate only showed a slight difference (23.49% in the low-risk group and 22.15% in the high-risk group) (Figures 7D,E). Coincidentally, in a melanoma cohort treated with immune checkpoint blockade (GSE91061), we found the risk score also distinguished the patients with clinical benefit (log-rank test between high- and low-risk group, *p* = 0.029, Supplementary Figure S3A). And the high-risk group showed significantly more non-responders (progression disease/stable disease (PD/SD) base on RECIST v1.1) compared to responders (partial response/complete response (PR/CR), Fisher exact test *p* = 0.011, Supplementary Figure S3B). We believe in the future, as the pancreatic cancer immunotherapy datasets are available, we can do further evaluation.

### Chemotherapy drug resistance and screening of potential therapeutic compounds

**FIGURE 8:**
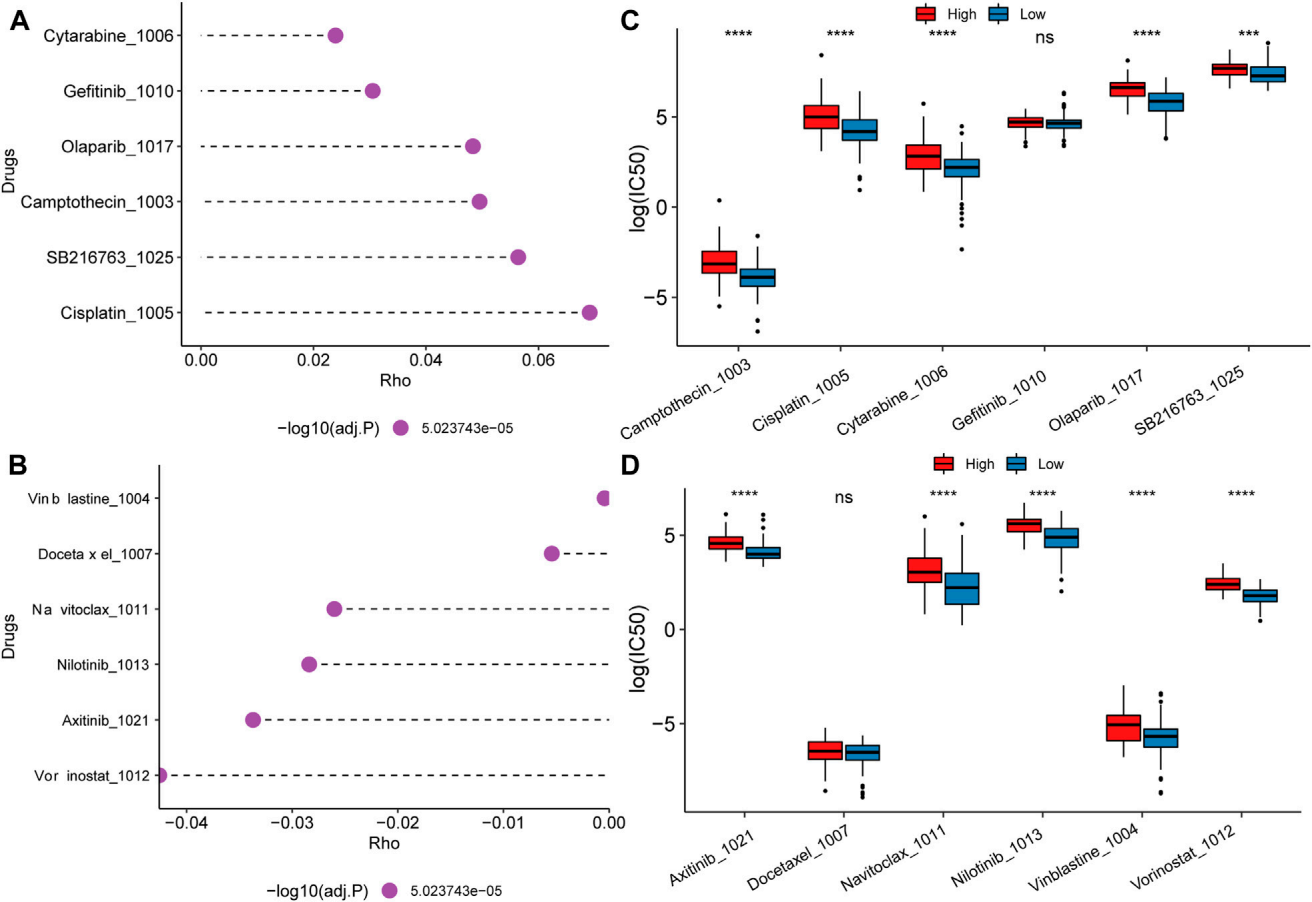
Drug resistance analysis and potential therapeutic compounds. **(A)** Top 6 drugs whose IC50 positively correlated with the risk score; **(B)** Top 6 drugs whose IC50 negatively correlated with the risk score; **(C, D)** IC50 distribution difference of positive and negative related drugs between high and low score groups.

Due to the worse prognosis of PAAD, we further investigate drug response and potential therapeutic compounds based on the mitochondrial energy metabolism-associated prognostic biomarker. Spearman correlation between half maximal inhibitory concentration (IC50) and the risk score reveled the top positive and negative correlated drugs (Figures 8A, B). For the positively correlated drugs, the IC50 of Cytarabine, Olaparib, Camptothecin, SB216763, and Cisplatin differed significantly between the high and low groups (Figure 8C). While for negatively related drugs, there was a substantial difference in the IC50 of Axitinib, Navitoclax, Nilotinib, Vinblastine, and Vorinostat between the high and low groups (Figure 8D). Findings proved that the model can be better applied to drug resistance analysis.

In addition, the study also screened potential therapeutic compounds using molecular docking based on the MMRG core genes. We selected lactate dehydrogenase LDHA with the highest prognostic performance as the docking protein and Cisplatin with the highest positive correlation with the risk score as the small molecule for molecular docking. Structure data of them were obtained from the PDB database and the ZINC small molecule database respectively. The autodock-vina software was used for molecular docking and Ligplus was used for interaction force analysis.

In the docking results, small molecules have some different cluster conformations, and the one with the lowest activation energy is selected for mapping. The docking affinity was −4.9 kcal/mol (Supplementary Figure S4). The models were of potential clinical value for potential therapeutic compound screening and state simulation.

## Discussion

During tumorigenesis, mitochondria are essential in regulating cell proliferation and apoptosis. The major metabolic pathways associated with mitochondrial energy metabolism are glycolytic/gluconeogenesis, tricarboxylic acid cycle, oxidative phosphorylation, ketone body metabolism, and lipid metabolism. The final 32 MMRG genes related to the mitochondrial energy metabolism pathway were obtained for analyzing the landscape alterations of pancreatic cancer and clinical implications. Additionally, a prognostic risk model of the mitochondrial energy metabolic pathway in pancreatic cancer for predicting patient prognosis as well as guiding relevant immunotherapy was built (Table 2).

**TABLE 2:** Key resources table-Software and algorithms.

Software or algorithms	Source
R package TCGAbiolinks	https://bioconductor.org/packages/TCGAbiolinks/
R package IMvigor210CoreBiologies	http://research-pub.gene.com/IMvigor210CoreBiologies
R package clusterProfiler	http://bioconductor.org/packages/clusterProfiler/
R package limma	https://bioconductor.org/packages/limma/
R package glmnet	https://cran.r-project.org/web/packages/glmnet/
R package survivalROC	https://cran.r-project.org/web/packages/survivalROC/index.html
ESTIMATE	https://bioinformatics.mdanderson.org/estimate/
R package GSVA	https://bioconductor.org/packages/GSVA/
R package xCell	GitHub-dviraran/xCell: Cell types enrichment analysis
R package oncoPredict	https://github.com/maese005/oncoPredict

It was found that all MMRG core genes were significantly different between cancer samples and normal samples. This confirms that the screened genes play an important role in PAAD. Based on survival analysis, 6 out of 32 MMRGs (LDHA, ALDH3B1, LDHAL6B, PKM, ALDH3A1, and PGAM4) in pancreatic cancer were of survival significance. The enzyme lactate dehydrogenase A (LDHA), which is crucial for cell development, is typically overexpressed in tumor cells. Induced lactate dehydrogenase a supports aerobic glycolysis in activated T Cells (Peng et al., 2016). A fundamental mechanism for the control of LDH-A *via* acetylation was revealed in a work by Di Zhao et al., and they confirmed that LDHA K5 acetylation might be a potential marker of pancreatic cancer initiation (Fantin et al., 2006; Zhao et al., 2013; Ye et al., 2021). In a variety of cancers, including lung cancer (Moreb et al., 2008; Wei et al., 2015), breast cancer (Douville et al., 2009), and colorectal cancer (Chen et al., 2015), the enzyme aldehyde dehydrogenase 3B1 (ALDH3B1), which catalyzes the oxidation of aldehydes to the appropriate carboxylic acids, has been discovered to be important cancer stem cell marker. L-Lactate Dehydrogenase A-Like 6B (LDHAL6B) could activate L-lactate dehydrogenase activity and participate in the pyruvate metabolism process. A pyruvate kinase that acts as a catalyst for the transition of phosphate groups from phosphoenolpyruvate to ADP, creating ATP and pyruvate, is encoded by the Pyruvate Kinase M1/2 (PKM), which is involved in glycolysis. According to research, the lncRNA GACAT2 regulates osteophytes and mitochondrial activity in an inflammatory environment *via* binding to PKM1/2 proteins (Li et al., 2022). The metabolism of lipid peroxidation, corticosteroids, neurotransmitters, and biogenic amines all entail the oxidation of different aldehydes to their corresponding acids by the enzyme aldehyde dehydrogenase family 3 member A1 (ALDH3A1). The expression of ALDH3A1 in non-small cell lung cancer is high (Moreb et al., 2008). The protein, which is a part of the phosphoglycerate metastasis family involved in glycolysis is encoded by the phosphoglycerate mutase family 4 (PGAM4) gene. PGM is upregulated in breast cancer and has an impact on other cancers (Durany et al., 2000). According to our research, individuals with pancreatic cancer who were in the low-expression group had a much greater chance of surviving than those in the high-expression group, with the most significant LDHA being linked to glycolytic metabolism. Analysis of the mitochondrial energy metabolism pathway in pancreatic cancer revealed that it is mainly involved in the glycolytic gluconeogenesis process. This amplifies the importance of these six MMRGs that exhibit differential expression in the development of pancreatic cancer.

Univariable cox risk regression of 32 MMRG core genes yielded 14 differentially expressed genes, which were further modeled by LASSO Cox regression analysis to finally obtain 4 key prognostic genes: LDHA, ALDH3B1, ALDH3A1, and ADH6. LDHA, ALDH3B1, and ALDH3A1 are all connected to the genesis and development of various cancers and play a role in mitochondrial energy metabolism. Hepatocellular carcinoma exhibits downregulation of the ATP synthesis-related gene alcohol dehydrogenase 6 (ADH6), which is a key prognostic indicator for pancreatic cancer (Liao et al., 2017; Liu et al., 2020; Cao et al., 2022). These researches imply that the genes utilized to build risk models are tumor-related. In addition, based on ROC analysis in one, three, and 5 years of validation set and training set, the survival of the training and validation sets trended in agreement and the model had good utility. To further investigate the independence of the model, clinical traits and immune infiltration correlations were examined. Consistent with our previous findings, Clinical data between high- and low-risk groups for the presence or absence of chronic pancreatitis revealed substantial variations.

In summary, our study shows that the MMRG-based PAAD risk score model can be well used for patient staging as well as predicting the clinical prognosis of patients. Based on a series of bioinformatics analyses, pancreatic cancer is mainly associated with glycolytic gluconeogenesis, a pathway related to mitochondrial energy metabolism. Among them, LDHA, ALDH3B1, and ALDH3A1 have survival significance and exhibit potential clinical predictive and prognostic value.

## Conclusion

In this study, we investigated the dysregulated mitochondrial energy metabolism-associated pathways in PAAD and identified 32 MMRG core genes. They were altered among different clinical characteristic groups, and showed significant prognostic value and association with immune infiltration, suggesting critical roles of MMRG involve tumor genesis of PAAD. We constructed a four-gene (LDHA, ALDH3B1, ALDH3A1, and ADH6) prognostic biomarker based on univariate then LASSO cox analysis, then confirmed its efficiency and independence. Further analysis indicated the potential therapeutic compounds based on the mitochondrial energy metabolism-associated prognostic biomarker. Overall, we constructed a valid prognostic model for PAAD that can provide a reference for better clinical management of PAAD patients. (Table 2)

## Data Availability

The original contributions presented in the study are included in the article/Supplementary Material, further inquiries can be directed to the corresponding author.

## Supplementary material

SUPPLEMENTARY FIGURE S1 LASSO regression analysis of 14 potential prognostic factors. The abscissa represents the logarithm of the independent variable Lambda, and the ordinate represents the coefficient of the independent variable; **(B)** LASSO regression confidence interval of each lambda; **(C)** LASSO regression coefficient of key prognostic genes.

SUPPLEMENTARY FIGURE S2 Prognostic analysis of MMRG core gene signature in validation set. KM, ROC, risk score, survival time distribution, and expression of prognostic factors of MMRG core gene signature.

SUPPLEMENTARY FIGURE S3 Performance of the mitochondrial energy metabolism-associated prognostic biomarker in melanoma patients treated with immune checkpoint blockade (GSE91061). KM curve of the high or low-risk groups; **(B)** Objective response rate in high- and low-risk groups.

SUPPLEMENTARY FIGURE S4 Molecular docking results of Lactate dehydrogenase (LDHA) and Cisplatin.

## Abbreviations

AUC, Area under the curve; CNV, Copy number variation; GSEA, Gene set enrichment analysis; GTEx, Genotype Tissue Expression project; HR, Hazards ratio; IC50, Half maximal inhibitory concentration; LDHA, Lactate dehydrogenase; MMRG, Mitochondrial energy metabolism pathway-related gene; NK, Natural killer; OXPHOS, Oxidative phosphorylation; PAAD, Pancreatic cancer; PAAD, Pancreatic cancer; PPI, Protein-protein interaction; PPP, pentose phosphate pathway; ROC, Receiver operating characteristic; SNV, Single-nucleotide variation; ssGSEA, single sample gene set enrichment analysis; TCGA, The cancer genome atlas.
