# Supplementary figures and images for: Comprehensive analysis reveals signal and molecular mechanism of mitochondrial energy metabolism pathway in pancreatic cancer
Source: Front Genet. 2023 Feb 6;14:1117145. doi: 10.3389/fgene.2023.1117145 (PMC9939759; doi:10.3389/fgene.2023.1117145)

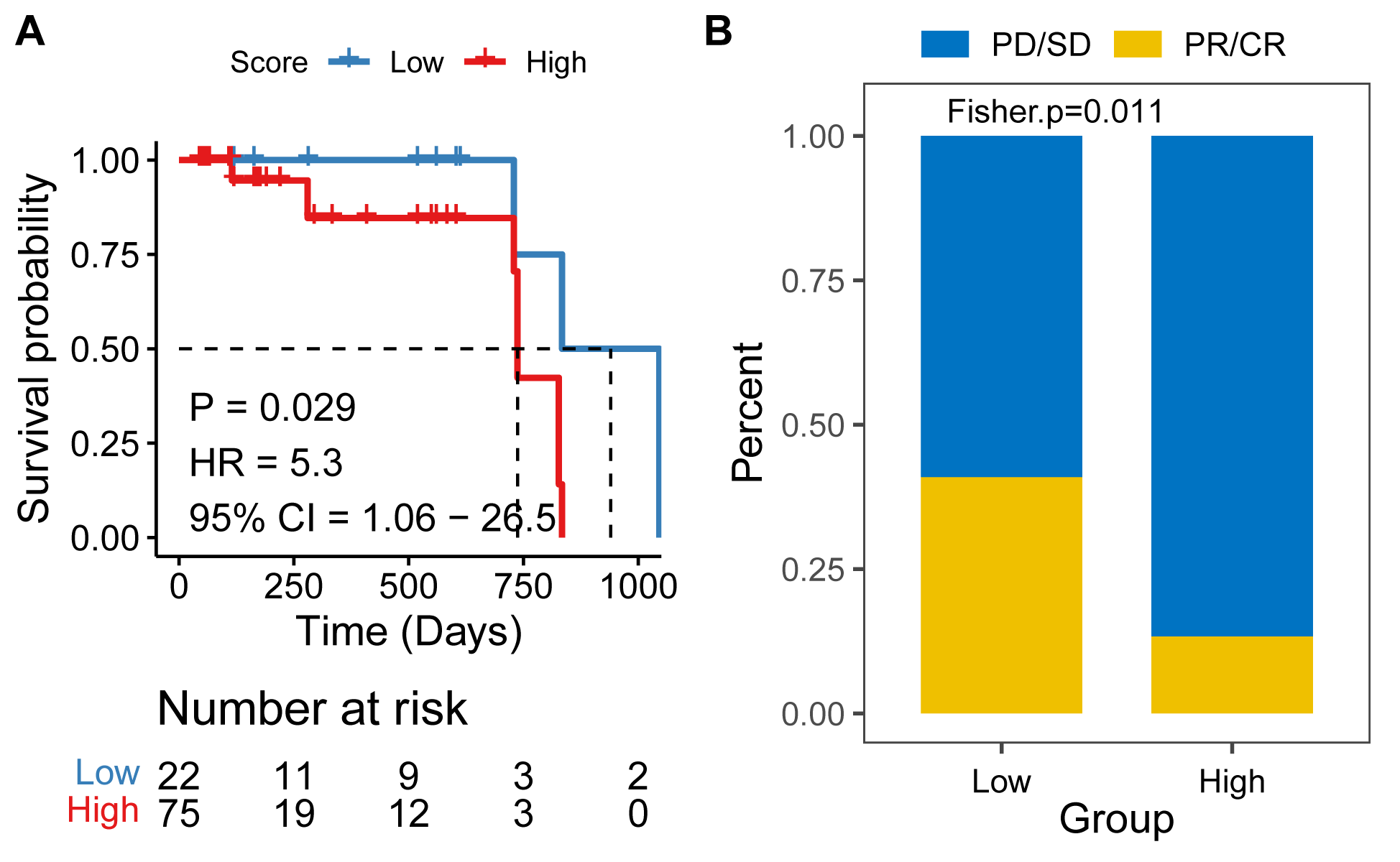

Supplement: Supplementary file 1 [file Image3.TIF]

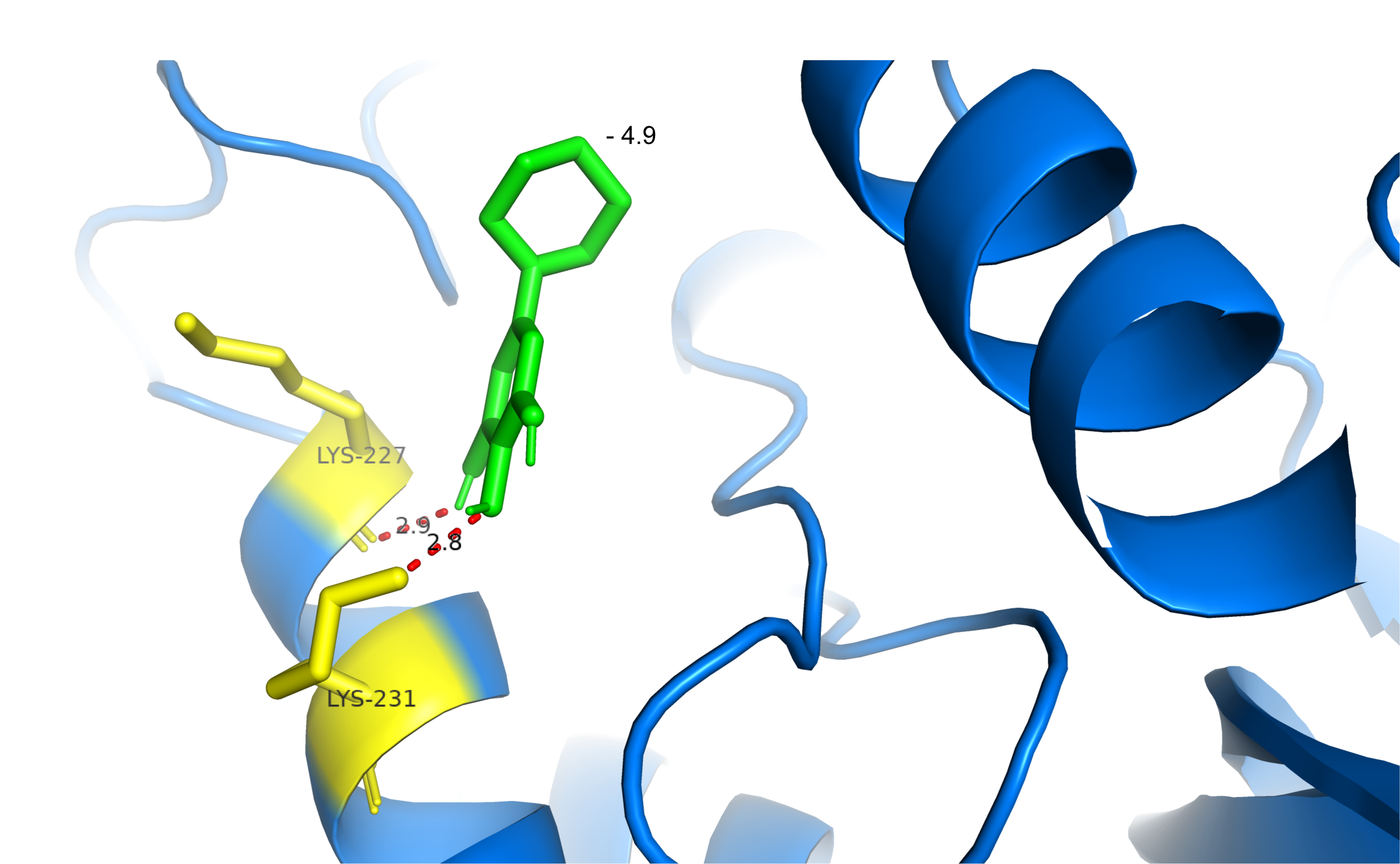

Supplement: Supplementary file 2 [file Image4.TIF]

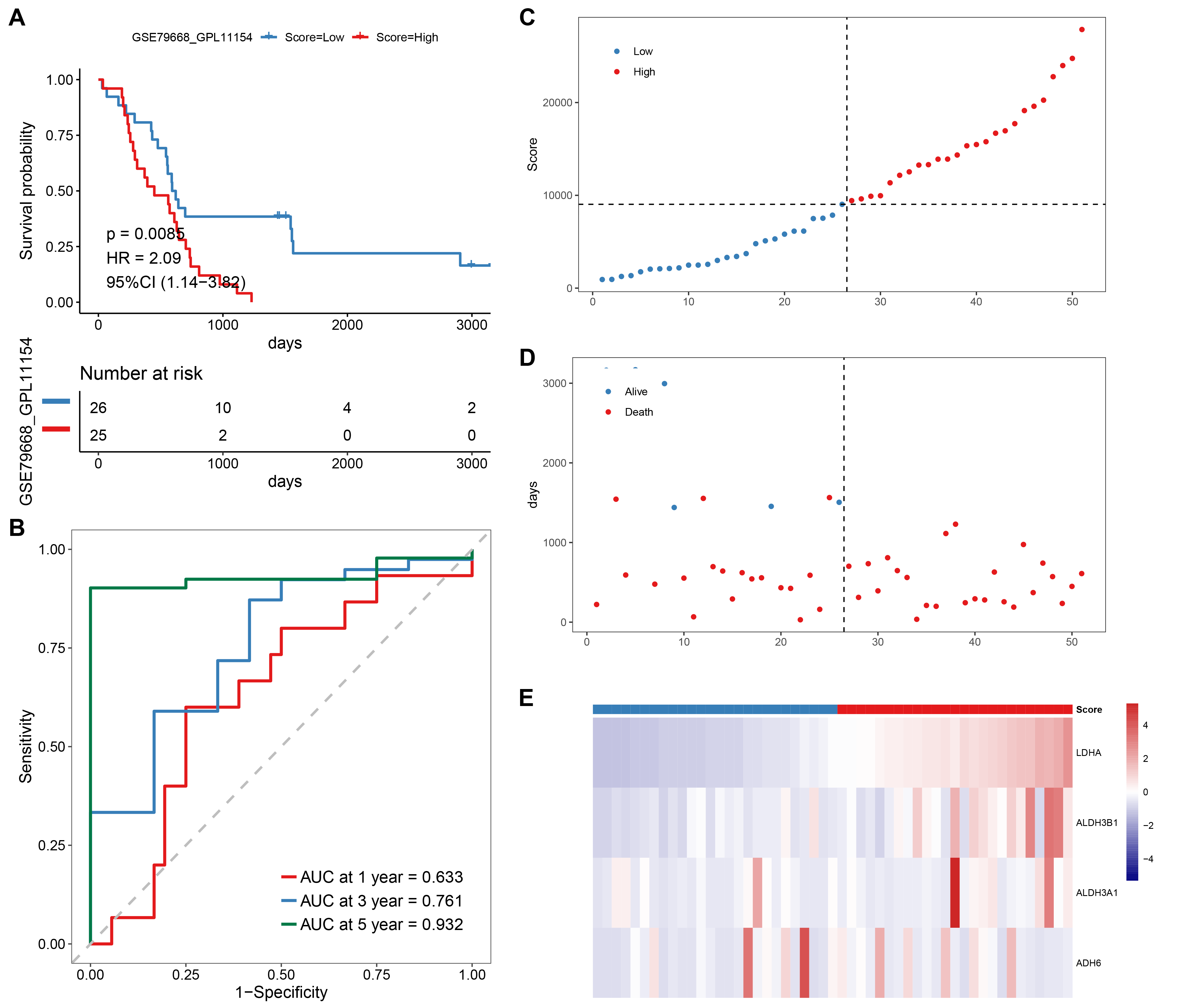

Supplement: Supplementary file 3 [file Image2.TIF]

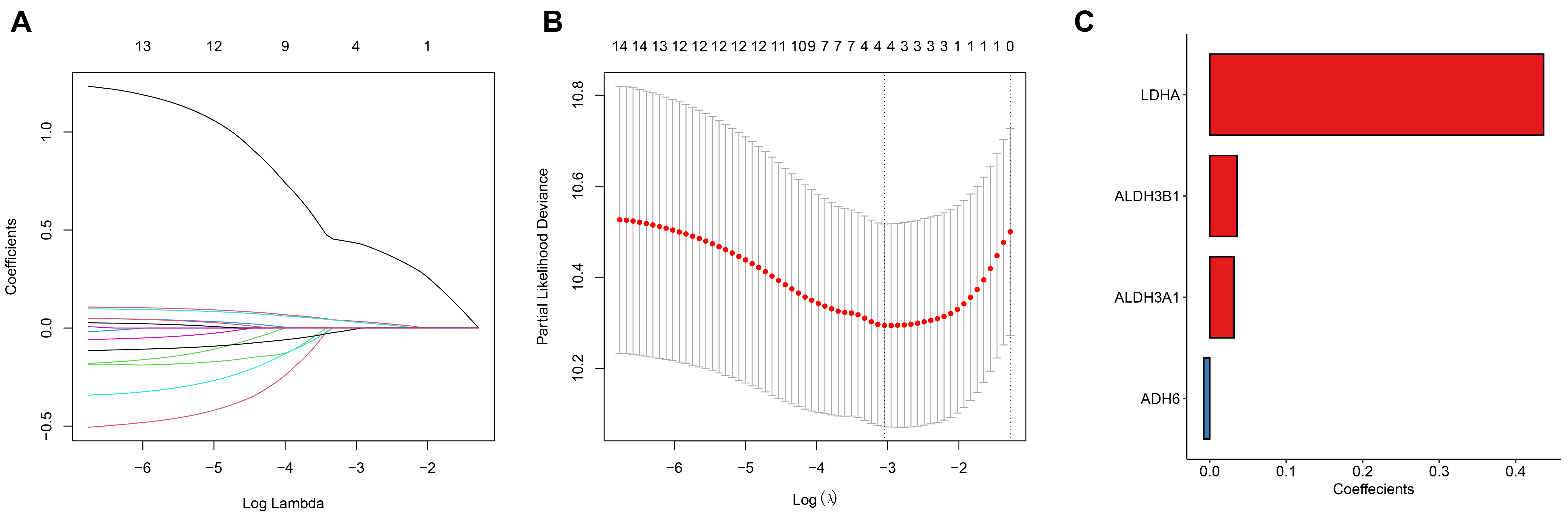

Supplement: Supplementary file 4 [file Image1.TIF]
